# Home Blood Pressure Monitoring by a Mobile-Based Model in Chongqing, China: A Feasibility Study

**DOI:** 10.3390/ijerph16183325

**Published:** 2019-09-10

**Authors:** Meng Xiao, Xun Lei, Fan Zhang, Zhenxing Sun, Vanessa Catherine Harris, Xiaojun Tang, Lijing Yan

**Affiliations:** 1 School of Public Health and Management, Research Center for Medicine and Social Development, Collaborative Innovation Center of Social Risks Governance in Health, Chongqing Medical University, Chongqing 400016, China (M.X.) (X.L.) (F.Z.); 2 Yuzhong Center for Disease Control and Prevention, Chongqing 400010, China; 3 Amsterdam Institute for Global Health and Development and Department of Global Health University Medical Center, location AMC, University of Amsterdam, 1105 Amsterdam, The Netherlands; 4 Global Health Research Center, Duke Kunshan University, Kunshan 215316, China

**Keywords:** mobile-based model, hypertension, feasibility, technology, self-management

## Abstract

*Purpose*: Increasing attention is being paid to the role of the intelligent self-management of hypertension under the context of increasing prevalence but limited medical resources. However, heterogeneity in interventions and outcome measures has hindered the interpretation of research evaluating mobile health technologies for hypertension control, and little study of such technology has been performed in China. *Objective*: This was a feasibility study aimed to understand patient and medical practitioners’ acceptance and experience of a mobile-phone based platform for the management of hypertensive patients. *Methods*: The model used behavioral incentives for daily blood pressure measurement and physician-facing prioritization of patients based on level of blood-pressure control. Patients were enrolled by purposive sampling. The platform was used for two-week blood pressure monitoring through WeChat, which simulated our future app. Qualitative interviews with patients and providers were conducted in time. *Results*: Twenty hypertensive patients and two providers were enrolled and used the platform throughout the two weeks. Patients reported daily home blood pressure monitoring to be simple, feasible and increased their health awareness. Specifically, patients self-reported that reminders, the daily frequency and time of monitoring, and positive reinforcement were important for maintaining adherence. Providers reported that they could manage patients more quickly and accurately, but reasonable feedback information was needed to avoid excessive increases in workload. *Conclusion*: The adoption of mobile-based technology to monitor patient’s blood pressure may provide a practical solution for managing patients in Chongqing, China. Patient health education and enhanced app functionality could improve patient compliance and satisfaction while reducing provider workload.

## 1. Introduction

Hypertension, or elevated blood pressure, is a major risk factor for cardiovascular disease and related deaths [1] though patients often have suboptimal blood pressure control. With an aging population and changes in people’s living habits, the prevalence of hypertension is increasing, but population-level control is hampered by limited medical resources, particularly in low- and middle-income countries [2,3,4]. In recent years, increasing attention has been paid to the role of self-management in the treatment and control of hypertension [5]. Good patient self-management can reduce blood pressure [6], which may result in a decrease in cardiovascular morbidity and mortality [7]. With the rapid development of technology, the intelligent self-management of hypertension offers new opportunities [8,9]. However, heterogeneity in interventions and outcome measures has hindered the interpretation of research evaluating mobile health technologies for hypertension control, and few studies of such technology have been performed in China [10].

We envisaged designing and testing a home-based self-management model for hypertensive patients in Chongqing, China, aiming to improve the efficiency in blood pressure control under the context of limited medical resources. Patients would complete their blood pressure measurements at home every day and the blood pressure values could be inputted into an application automatically thorough Bluetooth instead of entered by patients manually. WeChat is a social app commonly used in China, which is easy to operate and extremely popular among middle-aged and elderly people. According to official data released by WeChat, as of September 2018, there are 1082.5 million active users every month, and 63 million active users are over 55 years old [11]. We conducted this feasibility study using WeChat in order to understand user experiences with the daily home blood pressure measurements through a mobile-based model to refine our app to facilitate hypertensive patients self-management of their blood pressure, and explore an approach for primary care providers to provide patients with timely and tailored interventions.

## 2. Methods

Before this study, two tests (beta test one and beta test two) had already been completed in four living labs (Amsterdam, Netherlands; Chongqing, China; Nairobi, Kenya; Durham, USA). In beta test one, ten non-patient volunteers were recruited per lab and we gathered user experience on daily measurements as well as the reminder and behavior feedback messages. In beta test two, two or three care providers per living lab were interviewed to identify essential features of the provider’s dashboard and evaluate the proposed user flow (Supplementary material 1).

In this study, we monitored patients’ blood pressure regularly using WeChat to simulate our future app and provided feedback to health care providers using a dashboard designed by experts. After two weeks of monitoring, we interviewed patients and providers who participated in this process. This study was approved by the Ethical Committee of Chongqing Medical University (approval number: 2017004).

### 2.1. Study Sites and Sampling

Considering the operability of this feasibility study, we selected two districts close to Chongqing Medical University, which, at this stage, were relatively easier to coordinate and connect with our research sites. Two communities in Jiulongpo District and Nan’an District were purposively selected as our research sites. A sample of ten hypertensive patients was recruited by the provider at each site using the method of purposive sampling to ensure that our sample reflected a gender balance and a wide range of ages. Inclusion criteria were the diagnosis of hypertension without severe cognitive impairment and ability to use WeChat with a smart phone. Patients were informed of the study risks and included if they signed the informed consent. Subsequently, patients were provided, enrolled, and received training on home use of the electronic sphygmomanometer (OMRON HEM-7120). The participants’ basic characteristics, which were collected during the training session, included blood pressure at entry, treatment plan, and frequency of measuring blood pressure prior to joining the study. Based on the National Institute for Health and Care Excellence (NICE) guidelines [12] and a study conducted by Clarke M. et al. [13], a fourteen-day period was highly recommended for home blood pressure monitoring, so we eventually determined the study duration to be two weeks.

### 2.2. Monitoring Details

In the thirty minutes prior to obtaining a blood pressure measurement, patients are required not to do any strenuous exercise, drink coffee. or smoke. If possible, patients also need to urinate before measurements. Patients are supposed to rest for at least five minutes before the measurement. During the measurement, patients should sit at the table with their feet flat on the floor and their back against the back of the chair, then put arms at the heart level and relax them on the table. Patients should take off any clothes that are too tightly bound to the upper arm, and preferably take a measurement from the left arm. If the blood pressure measured in the right arm is higher or patients are not allowed to use the left arm for medical reasons, the blood pressure can be measured in the right arm. Patients are required to make sure that blood pressure is measured on the same arm each time. They should put the cuff on the left arm, the lower edge of the cuff should be three centimeters above the elbow bend, the instrument wire needs to be in a visual position and point to the middle finger, and the arm should tighten slightly—not too tightly or too loosely. Patients cannot clench their fists during the measurement. When the measurement is completed, patients need to record their values (systolic blood pressure and diastolic blood pressure) displayed on the instrument. They should measure their blood pressure twice again in the same way, with an interval of one minute, and record the values.

After obtaining their measurements, the results are sent to the research assistant. A double check was completed by two researchers together when blood pressure values were received and before the average values were sent to care providers. We handled false measures in two ways: if the three values of the day were obviously different, we would ask the patient to re-measure the blood pressure three times as soon as possible and record the results; or if the average value of the day was abnormal compared with the past few days, it would be handled by the care providers.

### 2.3. Intervention, Data Collection and Analysis

The two-week feasibility study was mainly carried out by a trained postgraduate medical student. A WeChat message to remind patients to measure their blood pressure was sent at a fixed time (nine o’clock) every morning. If patients did not provide feedback on their blood pressure after one hour, they would be reminded again (only once per day). The student collected the self-reported blood pressure values of patients (three measurements, one minute apart) from the two sites every day, calculated the average value of each patient and then prioritized average values according to a set of pre-defined criteria. The four categories conformed with the Chinese national hypertension and treatment guidelines [14]: Red—the last measured blood pressure was > 180 mmHg systolic/110 mmHg diastolic; Orange—the average blood pressure was 135/85~180/110 in the past week; Green—the average blood pressure was <135/85 in the past week; Gray—inactive group, patients in this category have not measured their blood pressure for more than three days in the past week. The student sent the categorized and prioritized dashboard to providers each evening, and providers delivered corresponding interventions to the patients accordingly. Before the two-week study, we provided training to the care providers and gave them a standardized guideline to ensure they understood our study and task well in advance.

After the two-week home blood pressure monitoring through WeChat, twenty patients and two providers were interviewed. The interviews were conducted in the offices of the two sites through individual in-depth interviews and were presided over by the professional investigators of the research team, with an observer and recorder, respectively. Before the interview, the interviewers were trained to ensure the interview process could be conducted according to a unified standard. Interviews were recorded after consent to ensure the comprehensiveness and reliability of the results.

**Figure 1:**
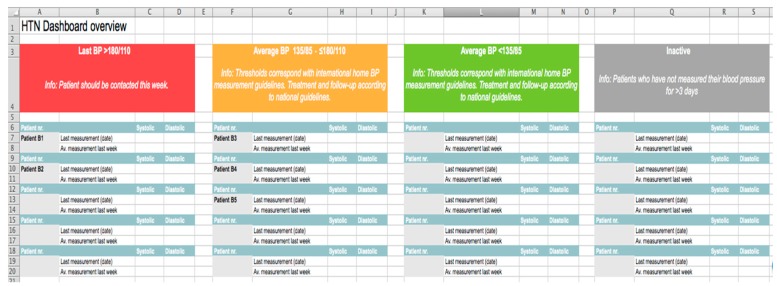
Overview of the dashboard.

**Figure 2:**
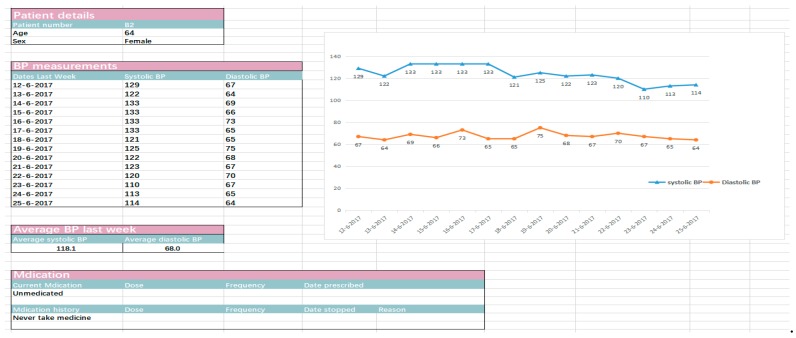
Template of the dashboard for a single patient.

A special group interview was organized at the two research sites, and each panel was organized by two researchers. One was responsible for questioning and guidance, and the other was responsible for observation and documentation. Participants, under the guidance of the organizers, were asked the same questions and expressed their opinions on different issues in turn. The focus group discussions (FGDs) were recorded with the consent of the participants, and each group discussion lasted approximately 30–40 min. The interview of patients was semi-structured. After discussion by experts, four key themes were addressed based on the “Guidelines for the Prevention and Treatment of Hypertension in China (2018 Revision)” [14] and beta tests from our two-week measurement, including “operational feasibility of blood pressure measurement”, “personal experience of home blood pressure monitoring in the past two weeks”, “factors affecting patients’ compliance with home blood pressure monitoring”, and “patients’ willingness to use an app to monitor blood pressure in the future”. Each key theme consisted of several relevant questions (Table 1). The interviews with the care providers at the two sites were conducted one by one based on our interview guide (Supplementary material 2), which aimed to acquire feedback from the care providers about their experiences with the Excel version of the dashboard (Figure 1 and Figure 2) used in this feasibility study.

After the interview, the recordings were transcribed verbatim by the local postgraduate medical students. Similar results were marked and a thematic framework for analysis and summary was established based on this. The transcribed results were encoded using Maxqda11 qualitative analysis software.

## 3. Results

### 3.1. Baseline Characteristics

Twenty patients were enrolled in the study and their ages ranged from 43 to 75 years old, with an average age of approximately 60 years old. Half of the patients were men. Six patients reported that they had never measured their blood pressure themselves before our study, six measured their blood pressure twice a month on average, three measured their blood pressure once a week on average, and the other five reported previously measuring their blood pressure daily (Table 2). We found significant differences (*P* < 0.05) in systolic blood pressure between the values we recorded when they were enrolled versus after one-week of monitoring (Table 3).

### 3.2. Interview Results

#### 3.2.1. User Experience and Feasibility of Home Blood Pressure Monitoring by a Mobile-Based Model in Patients

All of the patients indicated that it did not take long to complete the blood pressure measurements. Most of them (16/20) said it took less than five minutes, and none required more than ten minutes.

*“In less than five minutes. After the machine was tied to my hand all I needed to do was press it. Maybe it just (took me) two minutes.”*

(Nan’an, female, 48 years old)

*“It took (me) less than ten minutes to measure my blood pressure three times and average the values.”*

(Yangjiaping, female, 54 years old)

In addition, all patients thought the process of measurement was simple and the instrument was easy to operate, but two of them reported the process of measurement was a bit boring for them, even though the operation was not difficult.

*“It’s easy enough (for me to complete the measurement).”*

(Nan’an, female, 68 years old)

*“It’s easy to use the instrument. I just think the less trouble, the better. The process is a bit boring for me.”*

(Nan’an, male, 56)

Although the students used WeChat to remind patients to monitor their blood pressure every day during the study, some patients (7/20) indicated they did not recall the precise content of messages they received (including messages about daily measurement, the reminder when patients forgot to measure, or feedback on blood pressure, motivation after measuring, etc.). Patients who remembered the details showed a positive attitude toward the reminders:

“Every time I received the reminder, I felt grateful.”

(Yangjiaping, male, 61 years old)

“I am impressed with the message, (referring to the information on the phone), ‘You measured your blood pressure very positively yesterday, please stick with it tomorrow.’”

(Yangjiaping, male, 64 years old)

“It had me insisting to take the measurement. I had forgotten to measure my blood pressure at the set time somedays, and felt even a little guilty when I received the reminder again one hour later.”

(Yangjiaping, female, 64 years old)

All the patients expressed satisfaction with the length and tone of the messages they received. Four patients reported that it would be nicer if the messages could be diversified.

*“Well, I felt pretty satisfied. I think it was a concern for the elderly. The message wasn’t too long. No matter what you sent me, it was a motivation to us, which wasn’t annoying.”*

(Nan’an, female, 68 years old)

Fifteen of the twenty patients indicated that the two-week home blood pressure monitoring made them feel healthier. The other five patients did not report an impact on their self-perceived health—among them, four believed it was not necessary to measure blood pressure every day, as s/he felt that s/he would be okay if they just took their medicine, and one patient did not feel a change in health because he already had the habit of daily blood pressure monitoring.

*“I was in a better mood (with the measurement during the study), I also recorded my own blood pressure every day. I was really satisfied that my blood pressure was quite normal at this age.”*

(Nan’an, female, 75 years old)

*“At the very least, I knew more about my own blood pressure.”*

(Yangjiaping, female, 64 years old)

*“It had little effect on me. In fact, I used to measure blood pressure myself (before this study).”*

(Yangjiaping, male, 54 years old)

#### 3.2.2. Factors Affecting Patients’ Compliance with Home Blood Pressure Monitoring

Based on records from the dashboard during the two-week study interval, half of the twenty patients (10/20) performed daily measurements, six patients measured thirteen times, one patient measured eleven times, two patients measured ten times, and only one patient measured seven times during the study. 

When patients were asked about the reasons why they measured their blood pressure, the vast majority (19/20) said it was because they believed it was good for their health. Seven of them said they enjoyed the process, which was also one of the reasons why they performed the measurements. One patient indicated that measuring or not measuring their blood pressure was equivocal.

*“It’s not entirely because of the benefits, I also like the measurement.”*

(Yangjiaping, female, 64 years old)

*“Actually, I don’t think it is necessary to measure the blood pressure. I never felt uncomfortable even when my blood pressure was 160 or 170.”*

(Nan’an, male, 53 years old)

Most patients (17/20) believed it would be easier to stick to measuring at a flexible time every day rather than a set time.

*“I prefer to measure my blood pressure at six o’clock am every day, I usually begin to exercise at 6:30, so I have to take the sphygmomanometer with me every morning to ensure my blood pressure value could be fed back at the specified time these days.”*

(Nan’an, female, 75 years old)

*“I am more willing to measure my blood pressure from ten to eleven o’clock am, because I need to buy food earlier than this period, and then prepare lunch for my grandchildren.”*

(Yangjiaping, female, 72 years old)

*“I would like to measure earlier in the morning if I need to go out in the afternoon, but I prefer to measure my blood pressure in the afternoon if I am busy doing housework in the morning.”*

(Yangjiaping, female, 54 years old)

When asked about their views on daily vs. weekly blood pressure measurements over the two-week period, half of the patients (10/20) believed daily blood pressure measurements were better, while the other eight patients felt daily measurements were not necessary. Two patients were neutral and thought weekly or daily blood pressure measurements were both acceptable.

*“I think it’s better to measure blood pressure every day. The value can be different after two minutes.”*

(Nan’an, female, 75 years old)

*“In fact, I think it (blood pressure) should be measured every day. Since you gave me this instrument I have measured my blood pressure twice a day.”*

(Yangjiaping, female, 64 years old)

#### 3.2.3. Patients’ Willingness to Use an App to Monitor Blood Pressure in the Future

In the interview, we also asked patients for opinions about using a related app to monitor blood pressure in the future rather than using WeChat. None of them (0/20) thought using WeChat every day was a great challenge to them, but eight of them thought using an app which could provide feedback on blood pressure automatically without inputting the blood pressure manually would be better than WeChat. Another twelve patients felt using either WeChat or the app was feasible for them.

*“It will be more labor-saving to receive feedback automatically by an application, and it’ll be nice if we do not need to input our blood pressure ourselves.”*

(Yangjiaping, female, 54 years old)

*“Both (application or WeChat) works for me. Although I could not input my blood pressure values manually by typing, I could use voice to feed back the values through WeChat.”*

(Yangjiaping, female, 64 years old)

Most patients (14/20) clearly indicated their willingness to measure their blood pressure in the future (daily or weekly). They believed they developed a habit during the two-week intervention. Two patients indicated either daily or weekly measurement in the future were both acceptable for them.

*“I’d like to (measure blood pressure in the future), since I have developed the habit during these days.”*

(Nan’an, female, 75 years old)

*“Sure, I think it’s a good thing and a responsibility for our health.”*

(Nan’an, female, 68 years old)

#### 3.2.4. Health Care Provider Experience with Home Blood Pressure Monitoring Using a Mobile-Phone Model

Providers were in agreement across the two sites and they checked the dashboard daily and provided usual care to the patients based on the standardized guideline which was provided during training. After receiving the categorized and prioritized dashboard with patients’ information, they were most concerned about patients in the red and orange categories. Additionally, mean values and fluctuations of blood pressure were used as references for the treatment of patients.

*“Patients in the orange category were those whose blood pressure was not well controlled, so I mainly paid attention to this area. There were also a few patients in the red category. I tended to use the fluctuation of the blood pressure over one week (Figure 1) as the basis for treatment, rather than a single value.”*

(Care provider, Nan’an, female)

*“I started with the red category and especially cared about the details of those unusual values; I would look at their mean values and fluctuations of blood pressure, so that I could determine whether the high value was caused by improper measurement methods or exercise prior to measurement.”*

(Care provider, Yangjiaping, female)

*“If patients appeared in the red category, they would be called into the clinic for the treatment; otherwise, patients would be observed for another period of time, and would be notified to come into the clinic if the value was still abnormal.”*

(Care provider, Yangjiaping, female)

Both providers believed the dashboard was extremely concise and clear and could help them understand their patients’ blood pressure better. However, there were also some concerns:

“The management model of daily monitoring increased the communication time between the patients and I, particularly for patients with uncontrolled blood pressure. It would be quicker (to check the dashboard) if their blood pressure was well controlled.”

(Care provider, Nan’an, female)

“It would be better if a weekly average of patients in the orange category and daily high-risk situations (red category) of patients were reported in the dashboard, and I preferred patients’ blood pressure values be ranked from high to low.”

(Care provider, Yangjiaping, female)

Some demands which require future consideration:

“During this study, we did not provide feedback to patients in the green category (those with controlled hypertension), but they actually also hoped to receive the provider’s feedback, otherwise they may think it’s useless for them.”

(Care provider, Yangjiaping, female)

“It would be better if patients’ medication, lifestyle, and complications could be added to the received dashboard.”

(Care provider, Yangjiaping, female)

## 4. Discussion

Research in developed countries indicated that mobile technology could be regarded as an effective compliment to chronic disease management to some extent [15]. It is our understanding that similar studies have rarely been performed in Chongqing, China. Research conducted in other counties has shown a positive role of mobile technology in accessibility to disease management [16,17], and some systematic reviews proved the potential of apps in improving health outcomes among patients living with chronic diseases [10,18]. With the increasing coverage of smartphones in recent years, apps related to medical care have attracted researchers’ attention in China [19,20], and also provide the possibility for the remote management of patients with chronic diseases, such as hypertension. In our research, we found some positive impacts on our patients and saw the prospect of using an app through a smart phone to help care providers manage hypertensive patients. We found this mobile health hypertension intervention was well accepted by both patients and care providers, and it was feasible through an easy-to-use, concise and clear prototype, though our study also showed some shortcomings which need to be improved moving forward.

Hypertension is a major health threat in both developed and developing countries [21,22], and the adequate control and management of hypertensive patients is challenging in numerous settings [23]. Mobile medical technology has become an innovative tool for clinicians, as it can achieve continuous remote patient surveillance for health care providers when it is applied to chronic disease management [24]. Quite a few studies found it promising to provide secondary prevention and, for Cardiovascular Disease (CVD) patients, improve health though a mobile-based model such as text messaging [25,26,27]. This may be the main reason why we found some patients’ systolic blood pressure lowered after one-week of monitoring compared to the values we recorded when they were enrolled in this feasibility study. In China, making the most of this technology may offer a new avenue to monitor blood pressure in patients with hypertension, especially in the current situation of medical resource shortage.

After training, it was not difficult, and it did not take long for elderly patients with hypertension to use the measurement instrument. From this point of view, it was feasible for patients to complete blood pressure measurements at home.

Reminders were used to remind the patient to complete the blood pressure measurement. Even though the specific content of the reminders showed no difference in the patient’s measurement behavior in our study, our results suggest the importance of reminders should not be neglected and this result was similar to a previous study [28]. It might be more helpful if reminders could be diversified, which may improve patient satisfaction with the app in the future. 

A promising prospect was found based on the current results of our study: patients had a healthier attitude toward life and gained a better understanding of their blood pressure through the at-home daily measurements during the intervention. Our two-week study of blood pressure monitoring helped most patients develop a habit of monitoring their own blood pressure. Additionally, patients had a positive attitude towards using a similar app in the future, which showed it was feasible to use mobile medical applications in community health care settings in urban China to manage hypertension patients. 

We also found an important reason for patients compliance with measurements: the hope of improving their health. Hypertension, a chronic non-communicable disease, usually does not show any symptoms [29] and is therefore often neglected by patients. The interviews showed that some patients, whose systolic blood pressure was sometimes even 160 mmHg after taking medicine during the study, did not pay attention to their blood pressure. This may reflect patients’ lack of understanding of this disease. Providers can carry out appropriate publicity and education during their routine follow-up, which may be conducive to improving the compliance of patients in monitoring blood pressure and taking medicine on time [30]. Daily blood pressure monitoring may result in habit formation and allow patients to commit to their treatment regimen despite not feeling any symptoms from their disease.

For patients, it may make it easier for them to adhere to blood pressure measurements if they can make a plan according to their own schedule. For health care providers, this technology provided them with a dynamic and effective way to control patients’ at-home blood pressure measurements [31,32], which was similar to our study, and their workload can be alleviated if hypertension measurements are organized and presented with weekly averages, and with patients ranked from uncontrolled (red, daily) to controlled (weekly). This was taken into consideration in our next steps. 

Additionally, and in order to increase the acceptance of a future app for patients in the green category, we believe adding more modules and information may be beneficial such as including patients’ lifestyle, complications, etc. Also, in the patients’ feedback and results, it would be more helpful for providers to offer corresponding diagnostic opinions and suggestions based on the overall situation of the patients. Mobile applications are a useful tool to provide patients with interventions regarding daily behavior and nutrition to reduce blood pressure [33]. A suitable application may lead to a rise in adherence to treatment prescriptions and lesser clinical inertia [31,34], and short-term potential to reduce risk of this disease has been mentioned in a previous study [35]. Treatment guidelines provided by the app could contribute to an increase in patients’ inquiries into medical history or other questions related to their illness [36]. Further improvement in this area may be conducive to a long-term use of our app in the future.

## 5. Strengths and limitations

There were some strengths and limitations of this study. We collected app features and fine-tuned the messages during beta test one and two, in order to ensure patients’ satisfaction with our future app. For example, time intervals between daily reminders were increased to one hour and we found the tone of the messages needed to be modified in beta test one. Based on this and after much discussion, we designed our dashboard to contain personalized reminders and feedback messages for patients (Supplementary material 3). However, this study was a feasibility and acceptability study and therefore had a small sample size, only twenty patients and two providers were enrolled in this study, and most of our patients were under 65 years old, which may influence our results to some extent. More senior citizens should be enrolled in our future work to make our results more indicative. The vast majority of our designed reminders were not used during this period, which may be one of the reasons why some patients thought that the messages needed to be diversified in the future. Manually checking and verifying blood pressure can be time consuming, which is also a limitation of this study. Moving forward, results would be transmitted through Bluetooth instead of manual input by patients, and appropriate methods such as intelligent algorithms for prediction, which was used in a previous study conducted by Clarke M. et al. [13], would be implemented. Future studies are needed to measure the impact of similar mobile health applications on hypertension control and cardiovascular outcomes.

## 6. Conclusions

In summary, through this mobile health hypertension intervention, we were able to demonstrate the feasibility, uptake and acceptability of the mobile health application for both patients and physicians. As we shift to a digital age, the adoption of mobile-based technology may provide a new approach for monitoring and treating patient’s blood pressure. More testing, modification, implementation and measurement of the effects of such applications on actual blood pressure control are surely needed in order to address the increasing health care burden of hypertension.

## Figures and Tables

**Table 1:** The key themes from patients’ interviews during the feasibility study.

Themes	Questions
1. Operational feasibility of blood pressure measurement	How long do you need to measure your blood pressure every day?
	Do you feel the process of measuring or use of the instrument difficult for you?
2. Personal experience of home blood pressure monitoring in the past two weeks	What was the most impressive or favorite reminder that you received during the past two weeks and why?
	How did you feel during the past two-week measurement?
3. Factors affecting patients’ compliance with home blood pressure monitoring	What time of day do you usually measure your blood pressure?
	What are the reasons for why you persist in blood pressure measurement?
	Would it be easier for you to keep on measuring at the time chosen by yourself?
	Do you prefer a daily or weekly measurement?
4. Patients’ willingness to use an app to monitor blood pressure in the future	would it be better to use an app to automatically feedback blood pressure in the future?
	Would you like to measure your blood pressure every day in the future?

**Table 2:** The basic characteristics of the interviewees.

Basic Characteristics	Total	Nan’an	Yangjiaping	*P* Value
**Gender**				0.4076
Female (%)	10	6 (60%)	4 (40%)	
Male (%)	10	4 (40%)	6 (60%)	
Age, mean (SD)	59.55 (8.91)	59.90 (10.06)	59.20 (7.57)	0.8694
Systolic blood pressure at entry, mean (SD)	140.50 (17.57)	142.50 (20.96)	138.50 (13.05)	0.6328
Diastolic blood pressure at entry, mean (SD)	83.25 (10.98)	81.30 (9.55)	85.20 (11.92)	0.4537
**Frequency of self-measurements before this study (*n*)**				0.1079
Never	6 (60%)	4 (40%)	2 (20%)	
Once every two weeks	6 (60%)	4 (40%)	2 (20%)	
Once a week on average	3 (30%)	1 (10%)	2 (20%)	
Once/day	5 (50%)	1 (10%)	4 (40%)	

**Table 3:** Impact of the two-week measurement on patients’ blood pressure.

Variables	At Entry, Mean (SD)	After One-Week Measurement, Mean (SD)	*P* Value	At Entry, Mean (SD)	After Two-Week Measurement, Mean (SD)	*P* Value
**Systolic blood pressure**						
Nan’an	142.50(20.96)	130.80(13.66)	0.3022	142.50(20.96)	131.60(15.84)	0.3465
Yangjiaping	138.50(13.05)	124.20(5.89)	0.0470*	138.50(13.05)	126.60(13.11)	0.1005
Total	140.50(17.57)	127.50(10.51)	0.0451*	140.50(17.57)	129.10(13.96)	0.0746
**Diastolic blood pressure**						
Nan’an	81.30(9.55)	78.60(11.26)	0.6499	81.30(9.55)	79.40(9.86)	0.7344
Yangjiaping	85.20(11.92)	76.60(9.34)	0.2015	85.20(11.92)	77.60(13.79)	0.3037
Total	83.25(10.98)	77.60(9.81)	0.1883	83.25(10.98)	78.50(11.35)	0.2866

*: *P* < 0.05

## Supplementary Materials

The following are available online at https://www.mdpi.com/1660-4601/16/18/3325/s1, Figure S1: Product development update; Table S2: Interview guide: user experience with providers dashboard beta test 3; Table S3: Message bank.
