# Supplementary material for: Home Blood Pressure Monitoring by a Mobile-Based Model in Chongqing, China: A Feasibility Study
Source: Int J Environ Res Public Health. 2019 Sep 10;16(18):3325. doi: 10.3390/ijerph16183325 (PMC6765873; doi:10.3390/ijerph16183325)

Interview guide: user experience with providers dashboard beta test 3


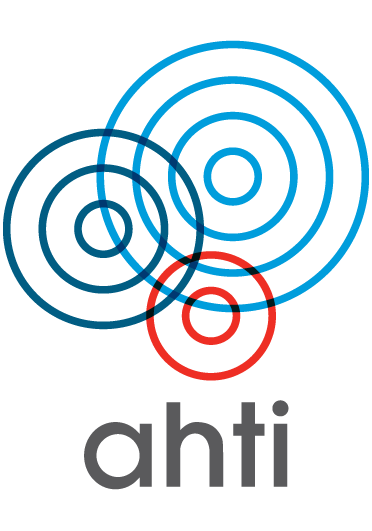


Instructions

Consider this document as a guideline for your interviews with the care providers, with the questions below as discussion topics.

**The aim of the interview is to get feedback from the care providers (doctors and nurses) about their experiences with the Excel-version of the dashboard used in beta test 3. The results of the interviews will be analyzed and used for further development of the prototype of the dashboard, which will be used in beta test 4. Preferably, the interviews will occur on a continuous basis by an in-person interview or by a phone call**. **This way, we can catch the learning curve of working with the dashboard. You can also divide the questions per interview, in order to make the interviews shorter. In addition, we advise a closing interview in order to retrieve concluding remarks.**

The structure can be as following:

1. Evaluation of the Excel-version of the dashboard
2. Recommendations for further development of the dashboard.

Please send ahti the transcription reports / summaries.

**Documents needed:**

- Interview guide
- Print 1: current Excel-version of the dashboard – Overview
- Print 2: current Excel-version of the dashboard – Patient file

**Evaluation of the Excel-version of the dashboard**

1. Have you used the dashboard on a daily basis?
2. What are your first experiences with the dashboard?
3. Do you understand the different categories in the dashboard overview?
4. Do you miss any information in the dashboard that you need to make an informed decision about the treatment and checkup frequency for the patient?

- In case you checked the current digital patient file in addition to the Excel Dashboard, what information did you check?

1. Did the dashboard influence your daily standard care for patients with hypertension?
2. How much extra time compared to the normal situation did you spend per patient (in minutes)?
3. Did you receive any feedback (both positive and negative) from patients about their daily home measurements?
4. Per column of the dashboard overview (red, orange, green, grey):
   - What actions did you take when reviewing the dashboard every day?
   - Did you contact any patients? If yes, in which column were they and what was the reason to contact them?
5. Per column of the dashboard overview (red, orange, green, grey):

- What actions would you take if you would work with the dashboard over a longer period of time?
- When would you contact a patient?

1. In the long run, we want to use this care model to monitor patients remotely and only invite those patients to the clinic that really need to see a doctor. A patient with a controlled blood pressure could for example be seen once a year. Would you feel comfortable with this? For which patients? Under which conditions?
2. If fully automated and connected to your existing digital patient file:
   - Would you use this dashboard in the future?
   - If yes, what do you like about the dashboard?
   - If no, what is needed for you to make the dashboard more useful?
   - Can you provide details on the level of integration with the existing patient file that you find ideal, i.e. which information should be automatically exchanged, do you believe this is possible?

**Recommendations for further development of the dashboard**

1. What information and/or features did you miss in the current Excel dashboard that should be included in the prototype?
2. Do you have any general recommendations for the dashboard prototype?

**Any other feedback**

Is there anything else you want to share with us? Any experiences that could help us to make our product better?

**Print 1: Dashboard-Overview**

**
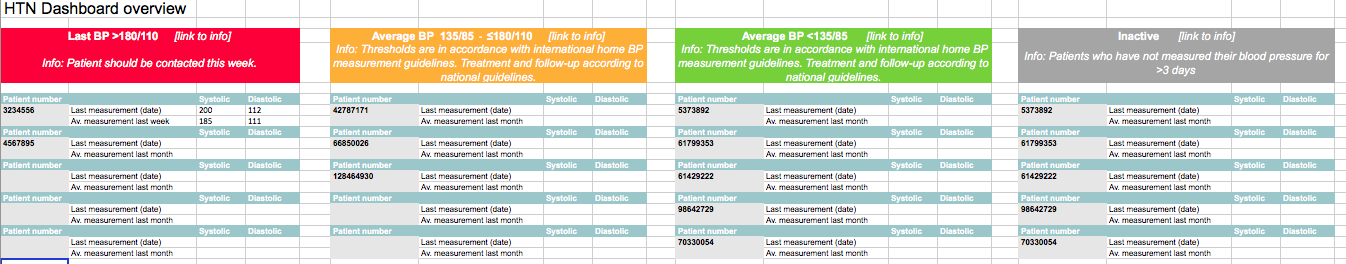
**

**Print 2: Dashboard-Patient file**


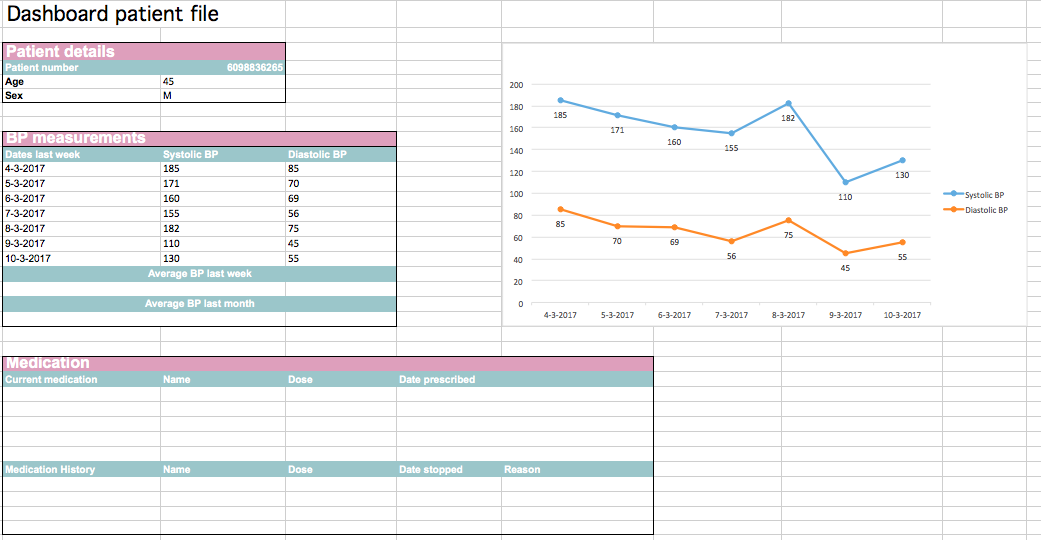

Supplement: Supplementary file 1 [file ijerph-16-03325-s001.zip › supplementary materials/supplementary 2.docx]
